# A promising “TRAIL” of tanshinones for cancer therapy

**DOI:** 10.7603/s40681-015-0023-8

**Published:** 2015-11-28

**Authors:** Tsing-Fen Ho, Chia-Che Chang

**Affiliations:** 1 Department of Medical Laboratory Science and Biotechnology, Central Taiwan University of Science and Technology, 406 Taichung, Taiwan; 2 Institute of Biomedical Sciences, National Chung Hsing University, No. 250, Kuo-Kuang Road, 402 Taichung, Taiwan; 3 Agricultural Biotechnology Center, National Chung Hsing University, 402 Taichung, Taiwan; 4 Ph.D. Program in Translational Medicine, National Chung Hsing University, 402 Taichung, Taiwan; 5 Rong Hsing Research Center for Translational Medicine, National Chung Hsing University, 402 Taichung, Taiwan

**Keywords:** Apoptosis, Cancer therapy, Danshen, Tanshinones, TRAIL

## Abstract

An ideal cancer therapy specifically targets cancer cells while sparing normal tissues. Tumor necrosis factor-related apoptosis-inducing ligand (TRAIL) elicits apoptosis by engaging its cognate death receptors (DRs—namely, DR4 and DR5. The cancer cell-selective proapoptotic action of TRAIL is highly attractive for cancer therapy, but clinical application of TRAIL is rather limited due to tumors’ inherent or acquired TRAIL resistance. Combining TRAIL with agents that reverse resistance to it has proved promising in the sensitization of TRAIL-induced apoptosis. Noteworthy, natural compounds have already been validated as potential resources for TRAIL sensitizers. In this review, we focus on the recently identified TRAILsensitizing effect of tanshinones, the anticancer ingredients of the medicinal plant *Salvia miltiorrhiza* (Danshen in Chinese). Research from our laboratories and others have revealed the synergy of a tanshinones-TRAIL combination in diverse types of cancer cells through up-regulation of DR5 and/or down-regulation of antiapoptotic proteins such as survivin. Thus, in addition to their anticancer mechanisms, tanshinones as TRAIL sensitizers hold great potential to be translated to TRAIL-based therapeutic modalities for combatting cancer.

## 1. Introduction

**Fig. 1:**
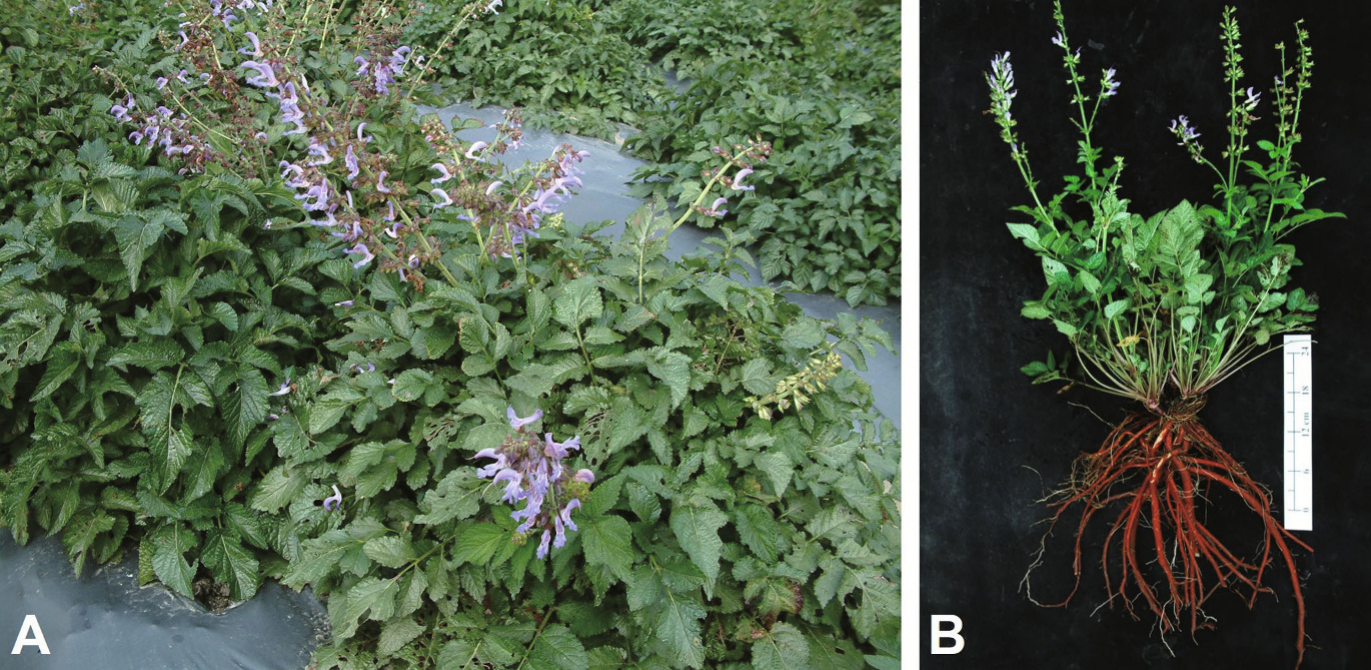
Photographs of *Salvia miltiorrhiza* Bunge (Lamiaceae). (A) Propagated plants of *Salvia miltiorrhiza*; (B) The aerial and root parts of harvested *Salvia miltiorrhiza*.

Cancer remains the leading cause of mortality globally. Despite advances in developing new therapeutic modalities for cancer, chemotherapy is still the fundamental tool for cancer treatment primarily through induction of apoptosis in cancer cells. Natural compounds isolated from medicinal plants have been seen as promising resources for novel chemotherapeutic drug discovery [1-3]. In this review, we summarize the anticancer potential of tanshinones, the bioactive components isolated from the dried root of the medicinal plant *Salvia miltiorrhiza* Bunge (Lamiaceae) (a.k.a. Danshen) (Figure 1) that has been frequently used in traditional Chinese medicine for over a thousand years to prevent or treat various conditions including menstrual disorders, hepatitis, and cardiovascular diseases [4, 5]. In particular, we focus on the recently discovered role of tanshinones as sensitizing agents of tumor necrosis factor (TNF)-related apoptosis-inducing ligand (TRAIL), which has an attractive anticancer potential due to its cancer cell-selective proapoptotic action but is often limited by the development of TRAIL-resistance in many human tumors. The mechanisms whereby tanshinones overcome TRAIL resistance and the potential translation of tanshinones to TRAIL-based cancer remedies are also discussed herein.

## 2. Tanshinones

### 2.1. Tanshinones are the anticancer components of Danshen

**Fig. 2:**
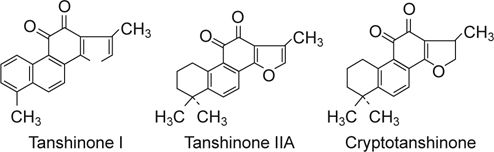
The chemical structure of the main tanshinones of Danshen. Tanshinone I (left); Tanshinone IIA (center); and Crytotanshinone (right).

**Fig. 3:**
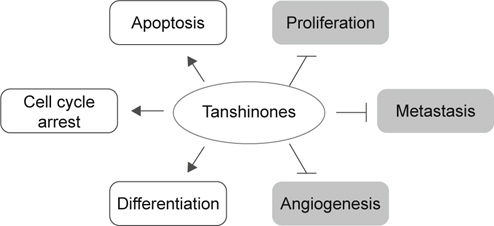
Anticancer mechanisms of action of tanshinones. Reported anticancer actions of tanshinones include: (1) inhibition of proliferation through arresting cell cycle progression, (2) induction of cancer cell apoptotic death, (3) anti-metastasis, (4) anti-angiogenesis, and (5) induction of cancer cell differentiation. Please refer to text for details.

In general, the bioactive components of Danshen can be categorized into two groups, namely, the lipophilic diterpene quinones and the water-soluble phenolic acids like salvianolic acids [6]. The lipophilic group, composed of more than 50 diterpenoid tanshinones, shows prominent anticancer potential in addition to showing anti-inflammatory and antioxidant activities [7]. Cryptotanshinone, tanshinone I, and tanshinone IIA are the three major elements of the lipophilic group (Figure 2), and numerous *in vitro* and *in vivo* studies have revealed the anticancer actions as well as the underlying mechanisms of these main tanshinones (Figure 3).

### 2.2. Anticancer modes of action of tanshinones

#### 2.2.1. Induction of cell cycle arrest

Tanshinones induce the arrest of cancer cell cycle progression at the G1, S, or G2/M phases in a cell type-dependent manner, leading to the inhibition of cell proliferation [8-12]. Mechanistically, tanshinone I has been shown to induce G1 arrest in lung cancer cells through the activation of the p53/p21/p27 pathway [13]. Cryptotanshinone and its synthetic derivatives as well as tanshinone IIA have all been observed to markedly repress prostate cancer cell growth *in vitro* and *in vivo* and to trigger G1 arrest by blocking the actions of the androgen receptor [11, 14-16].

#### 2.2.2. Induction of cell death

The proapoptotic effects of all of the main tanshinones have been tested and validated in a broad range of cancer cell lines, primarily through engaging the mitochondrial apoptosis pathway. Of note, all three main tanshinones have suppressed the activation of prosurvival STAT3 to provoke apoptotic cell death [17-20]. Furthermore, dependent on the type of tanshinones, additional prosurvival mechanisms have been found to be targeted for suppression, including PI3K/AKT [21, 22], survivin [23], Erb-2 [24], Aurora A [25], MCL-1, and c-IAP2 [26]. In contrast, activation of JNK [27], p53 [11], and endoplasmic reticulum stress have been reported to mediate tanshinones’ proapoptotic action [28, 29]. Intriguingly, the induction of autophagic cell death is something that contributes to the anti-leukemia effect of tanshinone IIA [30].

#### 2.2.3. Anti-metastasis

The anti-metastasis effect of tanshinone I has been clearly validated in xenograft models of the breast cancer cell line MDA-MB-231 [31] and the lung adenocarcinoma cell line CL1-5 [32], and has also been established in a transgenic lung cancer model driven by overexpression of the human vascular endothelial growth factor (VEGF)-A_165_ variant [13]. Additionally, tanshinone IIA inhibited the metastasis of xenografted hepatocellular carcinoma cell line HepG2, likely through the inhibition of the activities of matrix metallopeptidases 2 and 9 [33].

#### 2.2.4. Anti-angiogenesis

All of the main tanshinones demonstrate an anti-angiogenic effect at the *in vitro* and *in vivo* levels, as evidenced by reduced migration/proliferation/tube formation of vascular endothelial cells and neovascularization of the chick chorioallantoic membrane, respectively [18, 34, 35]. Tanshinone IIA has also been shown to repress angiogenesis in mice xenografted with MDA-MB-231 cells [36]. It appears that tanshinones elicit anti-angiogenesis mainly through the down-regulation of hypoxia-induced factor 1α (HIFα) and the consequent reduction in VEGF using distinct mechanisms. Tanshinone I lowered HIFα levels by promoting the proteasomal degradation of HIFα [18], whereas tanshinone IIA attenuated HIFα translation by suppressing the mTOR-p70S6K- 4E-BP1 signaling pathway [36].

#### 2.2.5. Induction of cancer cell differentiation

All-*trans* retinoic acid (ATRA) is an effective chemotherapeutic for acute promyelocytic leukemia (APL) that works by inducing APL cell differentiation, but resistance to ATRA does eventually develop. Notably, tanshinone IIA has effectively induced APL cell differentiation in both ATRA-sensitive and –resistant cell lines, likely through inducing CCAAT/enhancer-binding protein β (C/EBPβ)-mediated differentiation [37].

## 3. TRAIL

### 3.1. TRAIL-induced apoptosis

**Fig. 4:**
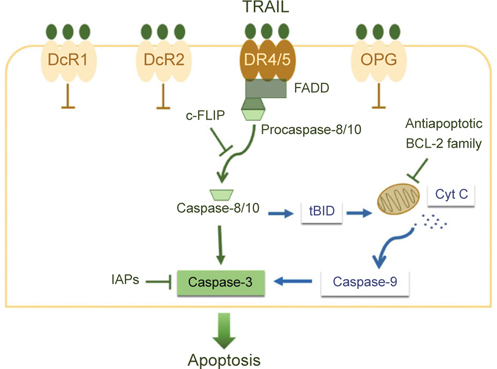
TRAIL-induced apoptosis signaling pathway. TRAIL initiates apoptosis through binding to DR4 and/or DR5. TRAIL binding induces receptor trimerization to promote the assembly of DISC (composed of FADD and procaspases 8/10) to induce self-cleavage and thus activation of caspases 8/10, which in turn trigger downstream caspase cascade to execute apoptosis program. In certain cell types, activated caspase 8 cleaves BID to generate truncated BID (tBID), which in turn triggers the mitochondrial apoptosis pathway. c-FLIP competes with procaspase 8 for recruitment to DISC, thereby suppressing activation of procaspase 8 and thus dampening TRAIL-initiated apoptosis stimuli. Antiapoptotic BCL-2 family members BCL-2, BCL-xL, and MCL-1 suppress the activation of the mitochondrial apoptosis pathway to blunt TRAIL-induced apoptosis. IAP proteins survivin and XIAP induce TRAIL resistance *via* the blockade of the activity of executioner caspases.

TRAIL is a type II membrane protein belonging to the TNF death ligand superfamily, which also includes TNFα and Fas ligand (FasL/CD95L) [38]. TRAIL is unique in its ability to induce p53- independent apoptosis selectively in cancer cells while sparing normal cells, thus avoiding the adverse side effects frequently associated with current chemotherapeutic agents. TRAIL induces apoptosis primarily through the death receptors (DRs)-mediated apoptotic pathway (Figure 4). Four membrane-bound TRAIL receptors, including DR4 (TRAIL-R1), DR5 (TRAIL-R2), decoy receptor 1 (DcR1/TRAIL-R3) and DcR2 (TRAIL-R4), and one soluble receptor osteoprotegerin (OPG) share highly homologous extracellular TRAIL-binding domain. Both DR4 and DR5 are functional TRAIL receptors that carry the cytoplasmic death domain (DD) to transduce TRAIL-initiated apoptotic signals, whereas DcR1, DcR2 and OPG lack the cytoplasmic DD and therefore antagonize TRAIL’s proapoptotic action. The binding of TRAIL to DR4 or DR5 induces receptor trimerization and consequently clusters the cytoplasmic DDs to recruit Fas-associated death domain (FADD) and pro-caspases 8 and 10 for the assembly of death-inducing signaling complex (DISC), leading to selfcleavage and thus activation of pro-caspases 8/10. Activated caspases 8/10 in turn initiate downstream caspase cascade to execute apoptosis program and, in certain types of cells, also evoke the mitochondrial apoptosis pathway through the truncation of the BH3-only protein BID (tBID) for the efficient induction of apoptosis [39].

### 3.2. TRAIL-based cancer therapy

The ability to induce cancer cell-selective apoptosis makes TRAIL an attractive choice for cancer therapy. Indeed, preclinical trials using soluble forms of recombinant TRAIL have shown a promising tumoricidal effect and, unlike TNFα and CD95L, barely caused systematic toxicity [39]. Clinical trials for TRAIL-based cancer therapies using either recombinant forms of the human TRAIL extracellular domain (dulanermin) or agonistic antibodies specifically targeting DR4 (mapatumumab) or DR5 (e.g. conatumumab) also revealed the safety and tolerability of these therapeutics. However, clinical trials of TRAIL-based therapies have failed to produce significant therapeutic responses in patients [38, 40, 41]. One of the key reasons for this limited therapeutic activity is TRAIL resistance, either inherent or acquired after repeated TRAIL administration.

### 3.3. TRAIL resistance mechanism

Our knowledge regarding the mechanisms of TRAIL resistance in tumor cells has advanced greatly in recent years. In general, the deregulation of the molecules involved in TRAIL-initiated apoptotic pathway is closely linked to the development of TRAIL resistance [41, 42]. Briefly, three fundamental mechanisms are commonly found in tumors with inherent or acquired TRAIL resistance. One, low levels of cell-surface DR4/DR5 and/or overexpression of DcR1/DcR2 effectively blunt TRAIL to turn on apoptosis. Two, TRAIL resistance can be caused by the upregulation of c-FLIP, a caspase 8 homolog without caspase activity; increased c-FLIP levels compete with procaspase 8 for recruitment to DISC, thus impairing the activation of caspase 8 to mediate TRAIL’s proapoptotic action. And three, overexpression of prosurvival proteins, including antiapoptotic BCL-2, BCL-xL and MCL-1 as well as inhibitors of apoptosis proteins (IAPs) such as survivin and XIAP, contribute to TRAIL resistance by blocking caspase activities.

### 3.4. Strategies to overcome TRAIL resistance

Given an acquired resistance to TRAIL commonly develops in most human tumors, current clinical trials for TRAIL-based therapies employ combination strategies using agents that overcome TRAIL resistance, thus restoring sensitivity to TRAIL-induced apoptosis [39-45]. Intensive studies in recent years have identified a number of potent TRAIL sensitizers in the context of diverse cancer cell lines. These include conventional chemotherapeutic drugs (e.g. cisplatin) [46], proteasome inhibitors (e.g. bortezomib) [47], Hsp90 inhibitors (e.g. 17-AAG) [48], ER stress inducers (e.g. tunicamycin) [49], and autophagy inhibitors [45]. BH3 mimetics and Smac mimetics, which selectively target antiapoptotic BCL-2 proteins and IAPs, respectively, synergize with TRAIL as well [50-53]. It is also noteworthy that some natural compounds have been validated as rich sources of TRAIL sensitizers [43, 44, 54, 55].

Reversing the mechanisms of TRAIL resistance forms the functional basis of TRAIL-sensitizing agents. Indeed, the majority of TRAIL sensitizers reported to date synergize with TRAIL by inducing up-regulation of DRs (particularly DR5), thus highlighting DRs’ expression levels as the primary point of control for TRAIL-induced apoptosis. In this context, DR5 is up-regulated through distinct mechanisms of action [43]. Most natural TRAIL sensitizers induce transcriptional up-regulation of DR5 in p53- dependent or -independent manners, the latter of which often involves the ROS-(JNK)-CHOP pathway, whereas the proteasome inhibitor bortezomib up-regulates DR5 by facilitating DR5 protein stabilization. Alternatively, caspase 8 activation caused by the down-regulation of c-FLIP at the transcriptional or posttranslational levels underlies the mode of action of some TRAIL sensitizers [45]. Likewise, small molecules that bind and stabilize the caspase 8 homodimers can function as TRAIL stabilizers by promoting caspase 8 activation upon TRAIL stimulation [56]. Chemotherapeutic drugs synergize with TRAIL mainly through lowering the apoptotic threshold by up-regulating proapoptotic BH3-only proteins while down-regulating antiapoptotic BCL-2 proteins and/or IAPs [39].

## 4. Tanshinones reverse TRAIL resistance

### 4.1. Tanshinones are a new class of natural TRAIL sensitizers

Using the TRAIL-resistant human ovarian cancer cell lines TOV- 21G and SKOV3 as cellular models, in early 2013 we published the first report demonstrating the TRAIL-sensitizing effect of crytotanshinones, tanshinone I, and tanshinone IIA [57]. Later on, Tse *et al*. identified crytotanshinone as a TRAIL sensitizer in the human melanoma cell line A375 and the lung adenocarcinoma cell line A549, both refractory to TRAIL [58]. Likewise, Shin *et al*. recently reported that tanshinone I restores the sensitivity of the TRAIL-resistant human prostate cancer cell lines PC-3 and DU145 to TRAIL-induced apoptosis [59]. These studies altogether establish tanshinones as effective TRAIL sensitizers. Noteworthy, up-regulation of DR5 appears as the fundamental basis of TRAIL sensitization by tanshinones. Detailed mechanisms whereby tanshinones overcome TRAIL resistance are summarized in the following sections.

### 4.2. Tanshinone IIA synergizes with TRAIL to induce apoptosis by engaging the ROS-JNK-CHOP signaling axis to up-regulate DR5 while activating p38 MAPK to down-regulate survivin

We made the pioneering discovery that crytotanshione, tanshinone I, and tanshinone IIA all exert TRAIL-enhancing action on the TRAIL-resistant human epithelial ovarian cancer (EOC) cell lines TOV-21G and SKOV3, with tanshinone IIA showing the best potency [57]. Subsequent analyses validated the synergy of this tanshinone IIA-TRAIL combination in apoptotic killing of these EOC cell lines, as well as the transcriptional up-regulation of *DR5* along with increased cell-surface DR5 expression following tanshinone IIA stimulation [60].

The levels of DRs on the cell surface is essential for TRAIL to induce effective apoptotic signaling, whereas low levels of cellsurface DRs confer TRAIL resistance. Along this line, the functional blockade of DR5 by the DR5/Fc chimer protein abolished tanshinone IIA’s action to sensitize TRAIL, indicating that DR5 up-regulation primarily determines tanshinone IIA as a TRAIL sensitizer in the context of EOC cells. Mechanistic studies on how tanshinone IIA up-regulates DR5 uncovered that tanshinone IIA triggers reactive oxygen species (ROS) production to induce JNK activation, leading to the transcriptional up-regulation of CAAT/enhancer-binding protein homologous protein (CHOP), a well-established transcriptional activator of the *DR5* promoter. The functional significance of the ROS-JNK-CHOP signaling axis in tanshinone IIA-mediated DR5 up-regulation is clearly supported by the failure of tanshinone IIA to induce DR5 when CHOP is depleted by CHOP shRNA, JNK-specific inhibitor SP600125, or ROS scavenger N-acetylcysteine (NAC) [60].

**Fig. 5:**
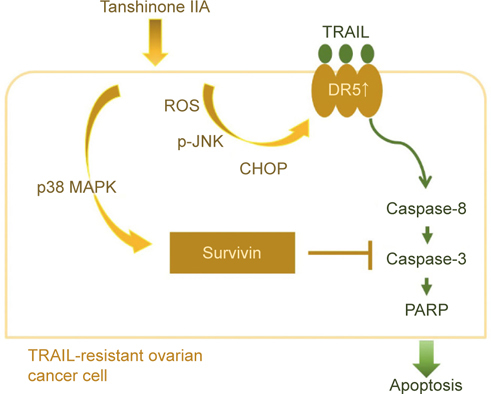
The mechanisms underlying tanshinone IIA-TRAIL synergy. Tanshinone IIA sensitizes TRAIL-resistant epithelial ovarian cancer (EOC) cells to TRAIL-induced apoptosis through at least two mechanisms. First, tanshinone IIA evokes ROS-dependent activation of JNK to increase CHOP expression, which in turn activate *DR5* transcription to up-regulate cell-surface DR5 levels for the potentiation of TRAIL-induced apoptosis stimuli. Second, tanshinone IIA engages the p38 MAPK-mediated pathway to induce transcriptional down-regulation of survivin, consequently promoting caspase activities to execute apoptosis.

Down-regulation of IAPs such as survivin or XIAP represents an alternative approach to overcome TRAIL resistance aside from DR5 up-regulation [42]. Survivin is recognized as an attractive drug target owing to its selective expression in malignant cells [61]. Notably, high levels of survivin have been associated with TRAIL resistance [62]. Along this line, we have revealed that tanshinone IIA induces p38 MAPK-dependent transcriptional repression of survivin in TRAIL-resistant but not TRAIL-sensitive human EOC cell lines [23]. Of note, ectopic survivin expression to counteract tanshinone IIA-induced survivin reduction markedly attenuates the synergistic cytotoxicity of the tanshinone IIATRAIL combination, indicating survivin down-regulation as an important mode of action for transhinone IIA to overcome TRAIL resistance. Collectively, these findings have delineated that multiple mechanisms of action, including DR5 induction and survivin repression, are involved in the tanshinone IIA-mediated sensitization of TRAIL-resistant human EOC cells to TRAIL-induced apoptosis (Figure 5).

### 4.3. Tanshinone I restores TRAIL sensitivity through microRNA 135a-3p-mediated up-regulation of DR5

Using the human prostate cancer cell lines PC-3 and DU-145 as cellular models, Shin *et al*. demonstrated that Tanshinone I synergistically sensitizes TRAIL-induced apoptosis in these TRAIL-resistant cells [59]. Furthermore, the tanshinone I-TRAIL combination up-regulated the mRNA and protein levels of DR5 and activated the *DR5* promoter, and this was accompanied by an increase in cell-surface DR5 levels. Not surprisingly, the depletion of DR5 severely lowered the potency of tanshinone I to enhance TRAIL-induced apoptosis, confirming the essential role of DR5 up-regulation in tanshinone I-mediated TRAIL sensitization. Furthermore, Shin *et al*. made a unique, noteworthy discovery that microRNA 135a-3p (miR135-3p) elicited by the tanshinone I-TRAIL combination accounts for the up-regulation of DR5, whereas the miR135-3p inhibitor attenuated the apoptosis induced by the tanshinone I-TRAIL combination. However, how miR135-3p up-regulates DR5 was not demonstrated in the report. Likewise, although tanshinone I evoked ROS production in these cell lines, the roles of ROS in miR135-3p induction and TRAIL sensitization by tanshinone I were not addressed either.

### 4.4. Cryptanshinone facilitates TRAIL sensitization by activating the ROS-CHOP-DR5 pathway

To employ DR5 up-regulation as the strategy of to overcome TRAIL resistance, Tse *et al*. identified cryptotanshinone, among tanshinone I, dihydrotanshione I, and tanshinone IIA, as the most potent tanshinones to induce DR5 expression in the TRAILresistant human melanoma A375 cells [58]. Similar to tanshinone IIA, cryptotanshinone was revealed to induce transcriptional upregulation of DR5 along with increased levels of cell-surface DR5 expression. DR5 up-regulation is essential for cryptotanshinone to overcome TRAIL resistance in the A375 cells, as DR5 depletion markedly abrogated cryptotanshinone-mediated sensitization to TRAIL-induced apoptosis. Similar to tanshinone IIA, cryptotanshinone elicited the ROS-dependent transcriptional induction of CHOP, leading to increased *DR5* transcription for enhancing TRAIL’s proapoptotic action. It is also noteworthy that p53 is not required for cryptotanshinone to up-regulate DR5, as evidenced by the comparable induction of DR5 in the HCT116 cell lines with or without p53 expression. As a matter of fact, the p53- independent nature of DR5 up-regulation by cryptotanshinone is advantageous, considering that the majority of human cancer cells are deficient in p53-mediated apoptosis.

## 5. Conclusions and perspectives

TRAIL, which benefits from its malignant cell-selective proapoptotic action, is an ideal cancer therapeutic agent, but its potential is ironically sabotaged by either intrinsic or acquired resistance commonly developed by tumor cells. A combination of TRAIL with agents that overcome TRAIL resistance mechanisms has been validated in preclinical and clinical trials as a promising strategy to boost TRAIL’s efficacy. In this regard, tanshinones as effective TRAIL sensitizers hold great potential to be included in TRAIL-based cancer therapeutic regimens. As for the TRAIL-sensitizing mechanisms of tanshinones, it appears that ROS-mediated DR5 up-regulation is the primary modes of action (except for transhinone IIA, which also targets survivin). It would be informative to decipher additional targets of TRAIL resistance mechanisms likely modulated by tanshinones in the context of various types of tumor cells, as a detailed molecular understanding of tanshinones-elicited TRAIL sensitization would allow for a rational design for more effective tanshinones-TRAIL synergy. Furthermore, although only cryptotanshinone, tanshinone I, and tanshinone IIA are validated as TRAIL sensitizers to date, additional members of tanshinones that are more effective to circumvent TRAIL resistance are likely present and remain to be discovered. It is also worth noting that the action of tanshinones as TRAIL sensitizers have been demonstrated only at the *in vitro* level so far. Therefore, preclinical validation of tanshinones- TRAIL synergy in xenograft or orthograft mouse cancer models is necessary for subsequent clinical trial design, which will hopefully lead to the future translation of tanshinones to TRAIL-based cancer therapy.

## Acknowledgments

This work was supported by grants from Central Taiwan University of Science and Technology, Taichung, Taiwan (CTU104-P- 16); National Chung Hsing University and Agricultural Research Institute, Council of Agriculture, Executive of Yuan, R.O.C. (NCHU-TARI 9904 and NCHU-TARI 10104); Taichung Veterans General Hospital and National Chung Hsing University, Taichung, Taiwan (TCVGH-NCHU997606); and The Ministry of Education, Taiwan, R.O.C. under the ATU plan. We thank Dr. Jui-Sheng Lai (Taiwan Agricultural Research Institute) for kindly providing the photographs of *Salvia miltiorrhiza*. We are also grateful to our students for contributing to the tanshinones-TRAIL synergy studies, and apologize for the literature of tanshinones not being included in this article due to limited space.

## Declaration of interest

The authors declare no conflicts of interest for this work.
